# The impact of entrepreneurial team psychological capital on innovation performance: The mediating role of knowledge sharing and knowledge hiding

**DOI:** 10.3389/fpsyg.2023.1133270

**Published:** 2023-03-13

**Authors:** Minling Chen, XueMei Mi, Jing Xue, Yi Li, Junran Shi

**Affiliations:** ^1^School of Economics and Management, Xi’an Shiyou University, Xi’an, China; ^2^School of Finance and Public Administration, Yunnan University of Finance and Economics, Kunming, China

**Keywords:** entrepreneurial team, psychological capital, knowledge sharing, knowledge hiding, organizational innovation climate, innovation performance

## Abstract

**Introduction:**

The important role of psychological capital on corporate innovation has attracted the attention of academics, more and more scholars have conducted related studies. Although most scholars have explored the mechanisms and paths of psychological capital on innovation performance, few scholars have explored the inner relationship between them from the perspective of knowledge management. Based on the knowledge management perspective, We explore the influence effect of the psychological capital of entrepreneurial teams on the innovation performance of startups in the entrepreneurial situation.

**Methods:**

We conducted hypothesis testing using data from 113 Chinese entrepreneurial teams, and conducted reliability analysis, correlation analysis, and regression analysis on the questionnaire data with the help of SPSS software and AMOS software.

**Results:**

The results showed that (1) entrepreneurial team psychological capital has a significant positive effect on innovation performance of startups; (2) entrepreneurial team psychological capital positively promotes their knowledge sharing behavior and reduces knowledge hiding behavior; (3) entrepreneurial team knowledge sharing and knowledge hiding play a partially mediating role between entrepreneurial team psychological capital and innovation performance of startups; (4)organizational innovation climate plays a moderating role in the influence of entrepreneurial team knowledge sharing and knowledge hiding on the innovation performance of startups.

**Discussion:**

The findings are consistent with the hypothesis model proposed in this paper, indicating that as the psychological capital of entrepreneurial teams increases, the innovation performance of startups can benefit from higher levels of knowledge sharing and lower levels of knowledge hiding.

## 1. Introduction

Driven by the severe employment situation and the strong entrepreneurial atmosphere, enterprises and individuals are increasingly willing to start their own businesses and are actively engaged in entrepreneurial activities. At the same time, with the introduction of the “mass entrepreneurship and innovation” policy, the competitive environment has changed, and enterprises must consider how to improve their own innovation performance. At the macro level, the improvement of innovation performance of entrepreneurial enterprises can greatly promote the dynamic development of regional and national economies, provide more and newer employment opportunities for the society, and become the engine of national economic growth (Wang R. et al., 2022); at the micro level, enterprises with excellent performance in innovation performance usually have a strong atmosphere of organizational innovation, which in turn motivates employees to engage in positive innovation behaviors. Therefore, how to improve the innovation performance of startups has become a hot topic at present, and scholars have conducted research based on internal and external factors affecting enterprise development, and found that psychological capital (PsyCap), as a positive psychological state shown by people in the process of work-life development, can effectively promote the rapid improvement of enterprise innovation behavior and performance. Given the importance of psychological capital in business development, more scholars have conducted research around the correlation between psychological capital and business performance, and the number of related literature published in high-level international journals is not few (Gao et al., 2020b; Grözinger et al., 2022). Although many scholars have continued to focus on psychological capital and business performance, after adding the element of “innovation,” the focus of scholars has been more on innovation ability and innovation behavior, and few scholars have directly focused on the relationship between psychological capital and innovation performance, which greatly affects people’s perception of the relationship between the two and creates some resistance to the innovation development of enterprises. At the same time, most scholars study psychological capital at the individual level, and few scholars raise the study of psychological capital to the team level, especially the lack of research on the psychological capital of entrepreneurial teams in entrepreneurial contexts. Therefore, this paper will investigate the process of “how psychological capital of entrepreneurial teams affects innovation performance of entrepreneurial firms in entrepreneurial scenarios.”

Psychological capital plays an important role in the process of business development, influence business performance directly or indirectly through various factors (Ngo, 2021; Jiao et al., 2022), task performance (Udin and Yuniawan, 2020; Al Kahtani and Sulphey, 2022), project performance (Zhang et al., 2022), safety performance (Peng et al., 2022), entrepreneurial performance (Gao et al., 2020a), and business performance (Chen and Tao, 2021) of the firm. With the increasing importance of innovation-driven strategy in enterprise development, the path to improve enterprise innovation performance has become a hot spot for scholars’ research, and psychological capital as a variable affecting innovation performance has been included in scholars’ analytical models (Guo et al., 2020), mediated by the intrinsic motivation of the transformational educational environment, and shown through empirical studies that workers’ psychological capital is positively related to innovation performance (Tran et al., 2021). Numerous scholars have focused on the impact of psychological capital on the performance of work, tasks, and innovation in firms, but few scholars have paid attention to the impact of team psychological capital on the innovation performance of firms, especially the impact of entrepreneurial team psychological capital on the innovation performance of startups based on entrepreneurial scenarios needs to be studied in depth.

How psychological capital affects firm innovation performance, in addition to considering factors such as intrinsic motivation, personal creativity, and readiness for change. As a new management idea and method emerging in the era of knowledge economy, knowledge management integrates modern information technology, business management ideas and modern management concepts, and plays an important role in the development of enterprises. Previous studies have shown that a significant relationship between team psychological capital and knowledge management (Zhang et al., 2022) and there is also a significant relationship between knowledge management and innovation performance (Jing and Cisheng, 2021; Chang et al., 2022; Ge, 2022). Knowledge sharing and knowledge hiding as an important part of knowledge management, it is representative to study the relationship between them and psychological capital and innovation performance. Scholars have used psychological capital as a mediating variable in their models when examining the relationship between the role of abusive supervision, ethical leadership, and knowledge sharing, empirical results showed that psychological capital was positively related to knowledge sharing (Agarwal and Anantatmula, 2021; Goswami and Agrawal, 2022); Zhu used psychological capital as a mediating variable when exploring the relationship between perceived overqualification and knowledge hiding behavior, and the results showed that psychological capital has a negative effect on knowledge hiding (Zhu et al., 2022). By combing through the relevant literature, Dongling et al. (2022) found that knowledge sharing has a positive impact on firm innovation performance, and the empirical results show that member knowledge sharing in the era of big data significantly impact the innovation performance of eSports industry knowledge alliances (Yue et al., 2022); scholars have shown through their research that knowledge hiding as an independent or mediating variable has a negative effect on task performance (Tian et al., 2022), team performance (Miao et al., 2022), organizational performance (Wen and Ma, 2021), and innovation performance (Rong and Liu, 2021) of firms. In summary, psychological capital has a correlation with knowledge sharing and knowledge hiding, and at the same time, knowledge sharing, knowledge hiding and innovation performance also have a correlation, but the existing literature lacks an overall study of the above four variables, and the inner connection between the four needs to be explored in depth.

Amabile et al. (1996) believes that organizational innovation climate is not only perceived by organizational members, but also influences their creative behavior. Organizational innovation climate is a typical extrinsic motivation that can affect employees’ performance. In layman’s terms, organizational innovation climate represents the extent to which the organization supports and encourages employees to actively participate in innovation activities (Bock et al., 2005). most scholars consider organizational innovation climate as a theoretical framework at the organizational level, a psychological climate that indirectly affects innovation performance of firms, Organizational innovation climate has been found to be an effective predictor of employee creativity and organizational innovation (Hsu and Chen, 2017). Scholars have shown that organizational innovation climate can positively moderate the impact of perceived benefits and perceived risks on digital transformation (Tsai and Su, 2022). The higher the organizational innovation climate, the greater the positive impact of positive emotional climate on innovation performance, and conversely the weaker the positive impact of negative emotional climate on innovation performance (Dou et al., 2022). The moderating role of organizational innovation climate in the process of firm development is more significant, but little literature has focused directly on its role as a moderating variable affecting knowledge management activities and innovation performance.

Based on the above analysis, this paper studies the correlation between the psychological capital of entrepreneurial teams and the innovation performance of startups in the entrepreneurial context by referring to theories such as positive organizational behavior. Knowledge sharing and knowledge hiding are selected as the mediating variables, and organizational innovation climate is introduced as the moderating variable to form the research model of this paper. In the process of investigation and data analysis, online and offline questionnaires were used to obtain relevant data, Likert scale was used to measure the data, confirmatory factor analysis was carried out on the measurement items, and SPSS and AMOS were used to test the reliability and validity of variables. The empirical results show that psychological capital of entrepreneurial teams affects the innovation performance of startups, Knowledge sharing and knowledge hiding play a partial mediating role in the two, Organizational innovation climate positively moderates the effect of knowledge sharing on innovation performance of startups and negatively moderates the effect of knowledge hiding on innovation performance of startups.

This study attempts to make some contributions in the following points: first, the article investigates the influence of psychological capital of entrepreneurial teams on innovation performance of entrepreneurial enterprises under entrepreneurial scenarios, completes the research on psychological capital and innovation performance at the team level, enriches the relevant theories, laying the foundation for scholars to later study the psychological capital of entrepreneurial teams and the improvement of corporate innovation performance under entrepreneurial scenarios; second, partial mediating role of knowledge sharing and knowledge hiding in the relationship between psychological capital of entrepreneurial teams and innovation performance of startups verified, this study clarifies the inner connection between the four through empirical research, it is beneficial to promote scholars to continue to explore the relationship between psychological capital and innovation performance along the knowledge management perspective in subsequent studies; third, the moderating role of organizational innovation climate in knowledge sharing, knowledge hiding and innovation performance of startups is verified, which facilitates the participation of other moderating variables in the study of knowledge management and innovation performance.

## 2. Theoretical background and hypotheses development

### 2.1. PsyCap of entrepreneurial team and innovation performance

As a global socio-economic phenomenon, entrepreneurship and innovation activities have received widespread attention at both the theoretical and practical levels (Singh and Gaur, 2018). In previous studies, scholars have placed high emphasis on the individualism and heroism of entrepreneurs and leaders, however, the highly complex and changing nature of today’s innovation and entrepreneurship environment makes innovation and entrepreneurial activities increasingly dependent on the joint efforts of team members, and the vast majority of successful businesses today are built on collaboration and frequently outperform than they would alone, this means that the entrepreneurial team plays a key role in the development of startups (Bolzani et al., 2019). The importance of entrepreneurial teams in entrepreneurial ventures cannot be overlooked: almost 95% of entrepreneurial individuals choose to collaborate with others or intend to do so in the future (Ruef et al., 2003). In addition, the high degree of uncertainty and risk associated with innovative entrepreneurial activity means that a variety of external and internal factors need to be supported in the process of innovation and entrepreneurship. Scholars currently consider the impact of external factors such as new product development coordination (Zhang and Min, 2022), value modularity (Wang J. et al., 2022), and knowledge search (Wang and Wang, 2022) on innovation performance, and similarly internal factors such as personality, psychological empowerment (Zhang, 2022), psychological contract (Zhang, 2022), psychological capital (Waters et al., 2020; Dimas et al., 2022), and other factors on innovation activities and innovation performance have also gradually received attention from scholars.

Psychological capital refers to the positive inner traits and positive psychological states that individuals possess and exhibit, and its concept originates from positive psychology and positive organizational behavior (POB). Luthans first classified psychological capital into four dimensions: self-confidence, hope, resilience and optimism, and it has been widely accepted by academics (Luthans et al., 2003). At the individual level, individuals with high psychological capital have stronger beliefs about innovation, are full of enthusiasm, energy, curiosity, have a spirit of exploration, are more willing to think and accept new ideas, and more likely to have the willingness to innovate and conditions to improve their overall innovation capacity (Luthans et al., 2011). Individuals with high psychological capital have higher hope and self-efficacy (Luthans et al., 2007) and are more likely to see the positive side of innovation when faced with innovation risks and setbacks, and are more likely to regain confidence and actively seek solutions to problems (Luthans et al., 2003; Andersson et al., 2020). At the team level, psychological capital increases work engagement behaviors and levels in a supportive learning climate to promote team innovation (Peng and Chen, 2022). At the organizational level, psychological capital, as an intangible asset for startups and SMEs, can help organizations generate more innovative activities in the face of exogenous crises (Grözinger et al., 2022). Psychological capital has a positive and significant contribution to both innovation activities and innovation performance: according to McKenny et al. (2013) psychological capital is particularly important for innovation performance, business growth in SMEs. Abbas et al. found that psychological capital is positively related to innovation job performance and negatively related to job stress in a study of employees in various organizations in Pakistan (Abbas and Raja, 2015). All four dimensions of entrepreneurial psychological capital, including self-confidence, hope, resilience, and optimism promote technological innovation, business innovation, and thus improve business performance (Gao et al., 2020a). Leader psychological capital positively contributes to team psychological capital, which in turn improves team innovation (Tho, 2020; Waters et al., 2020). When studying the effect of supervisor’s supervisory style on graduate students’ innovation performance in a Chinese educational context, Yang et al. (2022) found that graduate students’ psychological capital played a fully mediating role between the two, indicating that graduate students’ psychological capital is positively correlated with their innovation performance. Based on psychological theory, Ge et al. (2022) found that the psychological capital of knowledge employees in Shihezi region of Xinjiang had a significant contribution to their innovation performance based on a study of knowledge employees. To sum up, in previous studies, the psychological capital of entrepreneurs and employees at the individual level has been widely concerned by scholars, but little literature has studied team-level psychological capital, especially the psychological capital of entrepreneurial teams, however, in the new competitive environment, the complexity of innovation and entrepreneurial activities is increasing, and the importance of entrepreneurial teams is subsequently highlighted, this paper argues that in the entrepreneurial context, the psychological capital of entrepreneurial teams capital has a significant contribution to the innovation performance of startups, whereby hypothesis 1 is proposed:

H1: Psychological capital of entrepreneurial teams has a positive impact on innovation performance of startups.

### 2.2. The mediating role of knowledge sharing

Knowledge is an individual’s knowledge of things and is defined in Wechsler’s dictionary as the information, understanding, or skills that a person acquires from education or experience. Knowledge sharing is the willingness and behavior of individuals to share information about the learning process and new knowledge. Research has shown that psychological capital is an important factor in promoting knowledge sharing among employees (Qiu et al., 2015; Wu and Lee, 2017; Zhang et al., 2017). Maitlo et al. (2017) used public university researchers as respondents and the study proved that all four dimensions of psychological capital efficacy, hope, optimism, and resilience are related to knowledge sharing behavior of researchers in a university setting. Goswami and Agrawal (2022) confirmed that psychological capital has a positive contribution to knowledge sharing. In addition, collective psychological capital, based on individual psychological capital, describes collective members’ positive evaluation of the group’s environment and their expectations of collective development and success, including four dimensions of collective efficacy, hope, optimism, and resilience (Walumbwa et al., 2011). First, collective efficacy is the positive beliefs that collective members have in their work, which can help collective members overcome barriers to knowledge sharing and thus achieve knowledge sharing (Gully et al., 2002); collective hope is the collective members’ expectation of the organization’s plan to achieve a common goal, and collective hope can enhance collective members’ intrinsic motivation and promote their knowledge sharing behavior; optimism is the collective’s favorable evaluation of things in the work process, the members of a collective with optimism have a strong belief that the organization can achieve its desired goals, and rarely considers the adverse effects of communication and cooperation with others in its work, which is conducive to knowledge sharing (Peterson, 2000). A collective with high resilience can act quickly to find solutions to problems and is willing to help other collective members, making knowledge sharing among individuals sustainable (Coutu, 2002). Based on this, this paper argues that in an entrepreneurial environment, the psychological capital of entrepreneurial teams affects their willingness to share knowledge and behavior. That is, the psychological capital of the entrepreneurial team has the same positive contribution to its knowledge sharing, according to which, this paper proposes hypothesis 2.

H2: Psychological capital of entrepreneurial teams promotes knowledge sharing of entrepreneurial teams.

The psychological capital of an entrepreneurial team is a positive psychological state that affects the performance of the entire team when team members translate it into action. That is, the innovation performance of an entrepreneurial team requires team members to take the initiative to transform their positive psychological capital into concrete actions that are conducive to improving the team’s innovation performance in order to drive that team to achieve innovation performance in the end. Innovation is an activity in which knowledge is involved in a series of complex processes such as generation, transformation, and integration, and knowledge sharing promotes innovative behavior (Vandavasi, 2020; Derin et al., 2022; Xu and Suntrayuth, 2022). Knowledge sharing is the key to improve the innovation capability of a company (Saenz et al., 2009) and knowledge sharing behavior is a prerequisite for organizations to innovate (Del Giudice and Della Peruta, 2016). Moreover, knowledge sharing behavior leads to the generation of organizational innovation performance (Donate and de Pablo, 2015; Giampaoli et al., 2017). The generation of innovation performance inherently relies on tacit knowledge, and knowledge sharing facilitates the dissemination and flow of tacit knowledge in organizations (Nonaka, 1994; Del Giudice and Della Peruta, 2016). When studying the impact of new product development coordination on firms’ innovation performance, Zhang and Min (2022) suggested that knowledge sharing plays a mediating role between the two, it proved that knowledge sharing can promote enterprise innovation performance. Ritala et al. (2015) suggest that external knowledge sharing has a positive effect on innovation performance based on the results of a survey of 150 Finnish technology-intensive firms. Accordingly, this paper argues that teams with high levels of psychological capital are able to maintain the willingness to share knowledge with team members in the face of frustration and difficulties, thus obtaining the knowledge resources and key elements needed for team innovation and improving the overall innovation performance of the team. This paper proposes hypothesis 3.

H3: Knowledge sharing in entrepreneurial teams mediates the relationship between the psychological capital of entrepreneurial teams and the innovation performance of startups.

### 2.3. The mediating role of knowledge hiding

Knowledge management plays an important role in every organization, and it affects the performance of the whole organization, teams and individuals. Knowledge hiding is a separate concept that is in opposition to knowledge sharing (Zhao et al., 2019). The existence of “knowledge hiding” behavior makes it difficult to implement knowledge collection and integration in organizations. Previous studies have shown that employees are reluctant to share knowledge with others mainly out of defensive consciousness, trying to protect and control their knowledge ownership (Huo et al., 2016). According to the survey, about 50% of employees in organizations have the intention to retain, mislead, or conceal knowledge that others need when interacting with other members (Peng, 2013). Through scholars’ research on the antecedent variables of knowledge hiding, it was found that mainly the characteristics of knowledge, organizational-level factors, team and interpersonal factors and individual-level factors influence workers’ knowledge hiding behaviors, among which, the characteristics of knowledge include the complexity of knowledge itself and task relevance among colleagues; organizational-level factors include organizational rules, policies, knowledge management systems, knowledge sharing culture, etc.; team and interpersonal factors include team motivational climate, top management constraints, interpersonal equity, etc.; and individual factors include personality, self-efficacy, and goal orientation (He et al., 2021). Employees with high emotional intelligence are less likely to develop knowledge hiding behaviors because employees or team members with high emotional intelligence focus on teamwork and are more likely to build trusting relationships with partners in that team or organization than others (Xiong et al., 2021). Zhu et al. (2022) believe that individuals’ knowledge hiding behaviors may cause huge economic losses to organizations, and their research proves that the improvement of individuals’ psychological capital will reduce their knowledge hiding behaviors, thus bringing positive effects to organizations. This paper argues that in entrepreneurial teams, knowledge hiding behaviors among team members can hinder the benign development of the whole team, and the psychological capital of team members is a key factor affecting their knowledge hiding behaviors, i.e., the psychological capital of entrepreneurial teams reduces the knowledge hiding behaviors of team members. Accordingly, hypothesis 4 is proposed.

H4: Psychological capital of entrepreneurial team has a negative effect on knowledge hiding of entrepreneurial team.

Rong and Liu (2021) investigated the impact of knowledge hiding behavior on corporate innovation performance by using executive teams, and found that team knowledge hiding behavior is more complex than individual because, in addition to the psychological and cognitive factors of team members, interpersonal factors such as collaborative interactions of team members also affect team members’ knowledge hiding. In the long run, knowledge hiding at the team level can lead to a decrease in firm innovation performance (Chatterjee et al., 2021). Based on cultural dimension theory and social information processing theory, a multilevel linear model was used to analyze the data of university innovation teams, and the empirical results showed that knowledge hiding has a significant negative impact on the knowledge innovation behavior of university innovation teams (Zhang and Wang, 2021). Accordingly, this paper argues that in entrepreneurial teams, team members with high levels of psychological capital are able to collaborate more actively with team members in the cooperation process, which naturally reduces knowledge hiding behavior and thus improves the overall innovation performance of the team. This paper proposes hypothesis 5.

H5: Knowledge hiding in entrepreneurial teams mediates the relationship between the psychological capital of entrepreneurial teams and the innovation performance of startups.

### 2.4. The moderating effect of organizational innovation climate

Organizational innovation climate refers to the perception of organizational members on norms and behaviors that can promote the generation, development and realization of new ideas (Anderson et al., 2020). The interactive perspective argues that an individual’s environment changes his or her behavior. The innovation climate is a key factor for organizations to remain innovative and is the perception of organizational members about whether the organization encourages innovation and risk-taking; organizations with a strong innovation climate, organizational members possess openness and divergent thinking, and employees working in this climate are more willing to share knowledge (Munir and Beh, 2019). An organization that provides employees with a sense of security and creates an atmosphere where employees are not criticized for no reason is conducive to employees’ ability to think innovatively (Cabrera and Cabrera, 2005). An organizational environment in which employees feel comfortable encourages them to create and share knowledge (Fu et al., 2007). An innovative climate also empowers employees to think independently, and contribute to innovative performance by creatively reshaping their cognitive, motivational, emotional, and intellectual resources (Waheed et al., 2019). Empirical studies have examined the moderating effect of organizational innovation climate on different models. For example, Yu et al. (2013) have shown that organizational innovation climate not only directly affects innovation behavior but also increases the positive contribution of knowledge sharing to innovation behavior. Sung and Choi (2014) found that the positive relationship between interpersonal and organizational learning practices and innovation performance is stronger when the organizational innovation climate is higher. On this basis, we argue that organizational innovation climate provides a psychologically safe environment for organizational members to face challenges, exchange ideas, and encourage each other to learn and collaborate, and in the practice of team interest-oriented and more willing to share knowledge with team members, the frequency of knowledge sharing among members will increase, while the knowledge hiding behavior will subsequently decrease, and the overall innovation performance of the team is improved. Accordingly, this paper proposes the following hypothesis:

H6: Organizational innovation climate positively moderates the impact of knowledge sharing in entrepreneurial teams on innovation performance of entrepreneurial firms.

H7: Organizational innovation climate negatively regulates the influence of entrepreneurial team knowledge hiding on innovation performance of startups.

**FIGURE 1:**
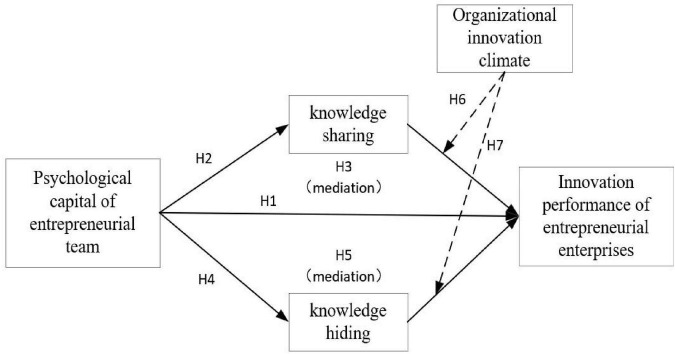
Theoretical model and research hypothesis.

In summary, the theoretical model of this article is shown in Figure 1.

## 3. Data and methods

In order to test whether the above hypotheses are valid and thus judge the rationality of the theoretical model, this study follows the scientific research paradigm and conducts empirical analysis according to the following steps: firstly, the questionnaire is designed with reference to the existing maturity scale, and the data are collected by distributing and collecting questionnaires; secondly, the reliability and validity tests are conducted by SPSS and Amos software to ensure the reliability and validity of the study; finally, SPSS 22.0 was used to conduct descriptive statistical analysis, correlation analysis and cascade regression analysis to complete the hypothesis validation and thus judge the rationality of the theoretical model.

### 3.1. Samples, data and processing methods

In this paper, a questionnaire was used, and the questionnaire was formed by translating and back-translating from foreign scales. An anonymous questionnaire designed with existing mature scales was used, and three discussions were held within the team to form the preliminary questionnaire. To ensure the accuracy of the questionnaire, three senior experts in related fields were invited to carefully check the content of the survey items. The questionnaire was collected from August to October 2022, 50 paper questionnaires and 100 electronic questionnaires were distributed, 137 questionnaires were returned, excluding invalid questionnaires that did not pass the reverse test set in the questionnaire, fixed answer multiple choice test questions, less than 300 s to fill in and scribble, 113 valid questionnaires were finally returned. Among them, 50 paper questionnaires were distributed in field research, 50 were collected, 50 valid questionnaires, with 100% efficiency; 100 electronic questionnaires were distributed, 87 were collected, 63 valid questionnaires, with 63% efficiency, and the total sample of valid questionnaires covered seven provinces such as Shaanxi, Sichuan, and Hebei.

The sample consisted of 113 entrepreneurial teams. The years of entrepreneurship ranged from 3 to 20 years, and most of them were in the range of 3–5 years (42.5%). Our selected survey respondents mainly include the initial founders (14.6%), board members (28.5%), professional advisors (22.4%), and core members (34.5%) of the startup. The size of the startups was indicated by the number of people in the business, ranging from 20 to 200. Among them, 58.5% are male and their age ranges from 21 to 58 years old. The majority had an educational background of bachelor’s degree or higher (76.9%). Specific basic information is shown in Table 1.

**TABLE 1:** Sample descriptive statistics.

Characteristic	Category	Percentage
Gender	Male	58.5%
	Female	41.5%
Age	<25	11.5%
	25–30	29.4%
	31–35	23.8%
	36–40	21.6%
	>40	13.7%
Educational background	Junior high school and below	5.7%
	High school	17.4%
	Junior college	24.6%
	Undergraduate	37.8%
	Master or above	14.5%
Years of entrepreneurship	<3	23.7%
	3–5	42.5%
	6–10	15.8%
	11–20	9.7%
	>20	8.2%
Team size	<20	31.8%
	21–50	27.1%
	51–100	15.9%
	101–200	16.3%
	>200	8.9%
Survey respondents’ positions in start-up companies	Initial founders	14.6%
	Board members	28.5%
	Professional advisors	22.4%
	Core members	34.5%

### 3.2. Measure of main variable

The psychological capital of entrepreneurial teams, innovation performance of startups, organizational innovation climate, knowledge sharing and knowledge hiding research variables involved in this paper were measured using a 5-point Likert scale (completely disagree = 1; completely agree = 5). Measures of psychological capital were first heavily applied at the individual level, and beginning around 2011, many scholars began to focus on psychological capital at the team and organizational levels (Walumbwa et al., 2011; McKenny et al., 2013) developed a Collective psychological capital measure. Based on previous research, we will use a revised team psychological capital questionnaire designed to assess the collective PsyCap of teams (Mathe-Soulek et al., 2014), and this study will measure four dimensions of entrepreneurial team psychological capital in an entrepreneurial context, namely team effectiveness, team hope, team optimism, and team resilience, with the final scale consisting of eight items. Each dimension is assessed by two items. Innovation Performance of Startups was measured using four indicators according to Fischer et al. (2001) and Zeng et al. (2010), and respondents were asked to indicate the extent to which they agreed with various statements about their firm’s innovation performance over the past 3 years when compared with competitors (see Table 2). Organizational innovation climate was measured using a 15-item innovation climate scale developed by Siegel and Kaemmerer (1978), modified by Scott and Bruce (1994), and well validated. Team knowledge sharing was measured using a questionnaire developed by Chuang et al. (2016), and the scale consists of seven items. We used two items developed by Serenko and Bontis (2016) and another two items developed by Connelly et al. (2012) to assess specific knowledge hiding behaviors. The specific measured entries for the five variables are shown in Table 2.

**TABLE 2:** Sample validity and reliability assessment of the measures.

Variable	Construct and measuring items	SFL
**Psychological capital of entrepreneurial team: α = 0.923; AVE = 0.705; CR = 0.950**		
PCET1	We are confident that we can solve the problems and difficulties that occur in the process of starting a business.	0.805
PCET2	We are very confident that we can achieve our goals in our own entrepreneurial field.	0.817
PCET3	Our team members can come up with many ways to achieve their goals.	0.792
PCET4	We feel we have achieved success in our work.	0.903
PCET5	We always see the positive side of the future development of the company.	0.891
PCET6	We always have an optimistic attitude toward our work.	0.782
PCET7	We are usually able to handle the stress at work with ease.	0.834
PCET8	We can get through the hard times at work.	0.882
**Knowledge sharing: α = 0.875; AVE = 0.626; CR = 0.921**		
KS1	Members of our team share their special knowledge and expertise with one another.	0.837
KS2	If a member in our team has some special knowledge about how to perform the team task, he/she will tell other members about it.	0.804
KS3	There is virtually no exchange of information, knowledge, or sharing of skills among members of the team (Reversed).	0.835
KS4	More knowledgeable team members freely provide other members with hard-to-find knowledge or specialized skills.	0.753
KS5	Members of our team provide a lot of work-related suggestions to each other.	0.747
KS6	There is a lot of constructive discussion during team meetings.	0.783
KS7	Members in our team provide their experience and knowledge to help other members find solutions to their problems.	0.774
**Knowledge hiding: α = 0.947; AVE = 0.668; CR = 0.889**		
KH1	In my project team, I often pretended that I did not know the information.	0.786
KH2	In my project team, I agreed to help my colleagues but never really intended to offer the knowledge they wanted.	0.791
KH3	I often communicated only part of the whole story to other project team members.	0.883
KH4	I often twisted the facts to suit my needs when communicating with other team members.	0.805
**Innovation performance of startups: α = 0.906; AVE = 0.584; CR = 0.848**		
IPS1	Proportion of annual turnover of new products.	0.801
IPS2	New products index.	0.674
IPS3	Modified products index.	0.739
IPS4	Patent growth rate.	0.832
**Organizational innovation climate: α = 0.897; AVE = 0.503; CR = 0.938**		
OIC1	Creativity is encouraged here.	0.747
OIC2	Our ability to function creatively is respected by the supervisor.	0.736
OIC3	Around here, people are allowed to try to solve the same problems in different ways.	0.705
OIC4	The main function of members in this organization is to follow orders, which come down through channels (Reversed).	0.725
OIC5	Around here, a person can get in a lot of trouble by being different (Reversed).	0.682
OIC6	This organization can be described as flexible and continually adapting to change	0.748
OIC7	A person cannot do things that are too different around here without provoking anger (Reversed).	0.646
OIC8	The best way to get along in this organization is to think the way the rest of the group does (Reversed).	0.765
OIC9	People around here are expected to deal with problems in the same way (Reversed).	0.634
OIC10	This organization is open and responsive to change.	0.628
OIC11	The people in charge around here usually get credit for others’ ideas.	0.757
OIC12	In this organization, we tend to stick to tried and true ways.	0.643
OIC13	This place seems to be more concerned with the *status quo* than with change.	0.672
OIC14	The reward system here encourages innovation.	0.787
OIC15	This organization publicly recognizes those who are innovative.	0.731

### 3.3. Control variables

We used these demographic characteristics as control variables, i.e., gender, age, and education, and in addition we added two control variables, years of entrepreneurship and team size, considering that this study is in an entrepreneurial context, for which we obtained relevant data in the survey items.

## 4. Data analysis

### 4.1. Test of common method deviation

In view of the possible problem of homogeneous bias caused by using the questionnaire form of data collection, this paper used Harman’s single factor analysis method to extract common factors for all entries of the five variables, and a total of five common factors were extracted with a cumulative explanatory power of 71.435%, and the explanatory power of the first factor was 24.198%, which was lower than the critical standard value of 40%. Therefore, it can be concluded that there is no serious problem of common method bias in this paper.

### 4.2. Reliability and validity test

First, this paper conducted validated factor analysis on the measured entries of all variables, and the results showed a KMO value of 0.945 and Bartlett test results passed the 0.000 significance level. Secondly, this paper conducted reliability and validity tests by SPSS and Amos software. According to the reliability test results in Table 2, the Cronbach’s α values of the reliability analysis of the four variables were higher than 0.8, and the combined reliability (CR) values of the latent variables were greater than 0.8 and greater than the critical value of 0.7, so it can be considered that the scale used in this paper has relatively good reliability and internal consistency. The results of the discriminant validity indicate a good five-factor model fit of the measurement model (CMIN/DF = 2.253 < 3, GFI = 0.914 > 0.9, RMSEA = 0.028 < 0.08, RMR = 0.031 < 0.05, AGFI = 0.933 > 0.9, CFI = 0.928 > 0.9, NFI = 0.924 > 0.9, and IFI = 0.917 > 0.9), the standard factor loadings obtained from the validation factor analysis were all greater than the critical value of 0.6, so the scale designed in this paper can be considered to have good discriminant validity. Finally, as the data in Table 2 show, the average variance extracted (AVE) values of all latent variables are greater than 0.5, and the flat square root of AVE is greater than the corresponding correlation coefficient between variables, so the scale can be considered to have good convergent validity.

### 4.3. Descriptive statistics and correlation analysis

The main variables in this paper include five. The means and standard deviations of the five variables and the correlation coefficients between the variables are shown in Table 3. The CFA results in Table 2 show that all standard factor loadings (SFL) values exceed 0.5, AVE values exceed the 0.5 threshold, and CR values for each construct exceed 0.6. Thus, the scale has good convergent reliability. As can be seen from Table 3, the square root of AVE of each construct exceeds the absolute value of the correlation coefficient between that construct and other constructs, indicating that the scale has good discriminant validity. From Table 3, it can be found that there are significant positive correlations among psychological capital of entrepreneurial team, organizational innovation climate, knowledge sharing and innovation performance of startups, and significant negative correlations between knowledge hiding and other variables, and the correlation coefficients are all below 0.5, indicating that there is no potential multiple co-linearity problem, and also providing a preliminary verification of the hypotheses.

**TABLE 3:** Descriptive statistical analysis and correlation coefficients.

Variables	Mean value	Standard deviation	1	2	3	4	5
Psychological capital of entrepreneurial team	4.013	0.644	1				
Knowledge sharing	4.134	0.519	0.298**	1			
Knowledge hiding	3.969	0.783	-0.254*	-0.459**	1		
Innovation performance of startups	3.768	0.607	0.323***	0.283**	-0.261**	1	
Organizational innovation climate	4.189	0.724	0.253***	0.304**	-0.247*	0.277***	1

***P < 0.001, **P < 0.01, *P < 0.05.

## 5. Hypothesis tests

### 5.1. Regression analysis on PsyCap of venture teams and innovation performance

Hierarchical regression was used to test the research hypotheses proposed in this paper, and the results of the hierarchical regression are shown in Table 4. Model 5 in Table 4 is the result of regressing the control variables of this paper-entrepreneur’s gender, age, entrepreneur’s education, years of startup founding and startup team size as independent variables on the innovation performance of startups, and Model 6 is the result of regressing the innovation performance of startups after adding the psychological capital of startup team to Model 5 Model 6 is the result of adding the psychological capital of the entrepreneurial team to model 5. Model 5 and model 6 show that the R2 of the model increases significantly after adding the independent variables, and the regression coefficient of psychological capital of entrepreneurial team on innovation performance of startups is 0.268, which is significantly positive at the 0.01 level, indicating that there is a significant positive effect of psychological capital of entrepreneurial team on innovation performance of startups, and hypothesis H1 is confirmed.

**TABLE 4:** Sample regression analysis of the impact of entrepreneurial team PsyCap on innovation performance.

Variable	Knowledge sharing		Knowledge hiding		Innovation performance of startups					
	Model 1	Model 2	Model 3	Model 4	Model 5	Model 6	Model 7	Model 8	Model 9	Model 10
Gender	-0.133	-0.156	0.109	0.143	-0.145	-0.132	-0.138	-0.133	-0.141	-0.131
Age	0.134	0.164	0.198*	0.157*	0.176	0.168	0.137	0.201	0.168	0.173
Educational background	0.113	0.134	0.141	0.137	0.129	0.124	0.125	0.109	0.121	0.125
Years of entrepreneurship	0.144	0.182	0.197	0.168	0.187	0.178	0.157	0.103	0.201	0.179
Team size	0.132	0.155	0.178	0.165	0.132	0.134	0.125	0.145	0.143	0.129
Psychological capital of entrepreneurial team		0.368***		-0.415**		0.268***	0.208**	0.237**	0.263**	0.257**
Knowledge sharing							0.329***		0.302**	
Knowledge hiding								-0.401**		-0.386**
Organizational innovation climate									0.304**	
Knowledge sharing × organizational innovation climate									0.267**	
Knowledge hiding × organizational innovation climate										-0.253**
*R*2	0.034	0.267	0.089	0.341	0.046	0.297	0.369	0.378	0.403	0.398
Adjusted *R*2	0.026	0.251	0.080	0.315	0.037	0.299	0.329	0.347	0.361	0.372
*F*	1.689	10.605	2.013	9.787	1.981	10.312	13.115	14.017	15.819	14.899

***P < 0.001, **P < 0.01, *P < 0.05.

### 5.2. A test of intermediary effect of knowledge sharing

To verify the mediating effect of knowledge sharing, Model 1 in Table 4 is the result of regressing the control variables of this paper-entrepreneur’s gender, age, entrepreneur’s education, years of startup founding and startup team size-on knowledge sharing as independent variables, and Model 2 is the result of adding the psychological capital of the startup team to Model 1 on startup Model 2 is the result of adding the psychological capital of entrepreneurial team to Model 1 and regressing the knowledge sharing. It can be found from model 1 and model 2 that the R2 of the model increases significantly after adding the independent variables, and the regression coefficient of entrepreneurial team psychological capital on knowledge sharing is 0.368 and significantly positive at the 0.01 level, indicating that there is a significant positive effect of entrepreneurial team psychological capital on knowledge sharing, and hypothesis H2 is verified.

Model 7 in Table 4 shows the results of regressing entrepreneurial team psychological capital and knowledge sharing into model 5 simultaneously on the innovation performance of startups. It can be found that the absolute value of the regression coefficient of entrepreneurial team psychological capital becomes smaller (from 0.268 to 0.208) and the significance level becomes lower relative to model 6 after the introduction of the mediating variable knowledge sharing, indicating that the mediating effect of knowledge sharing exists and is partially mediated the hypothesis H3 was verified.

### 5.3. The test of intermediary effect of knowledge hiding

To verify the mediating effect of knowledge hiding, Model 3 in Table 4 is the result of regressing the control variables of this paper-entrepreneur’s gender, age, entrepreneur’s education, years of startup establishment and startup team size on knowledge hiding as independent variables, and Model 4 is the result of regressing knowledge hiding by adding the psychological capital of the startup team to Model 3. Model 4 is the result of regressing knowledge hiding by adding entrepreneurial team psychological capital to model 3. Model 3 and model 4 show that the R2 of the model increases significantly after adding the independent variables, and the regression coefficient of entrepreneurial team psychological capital on knowledge hiding is 0.415 and significantly negative at the 0.05 level, indicating that there is a significant negative effect of entrepreneurial team psychological capital on knowledge hiding, and hypothesis H4 is verified.

Model 8 in Table 4 shows the results of regressing entrepreneurial team psychological capital and knowledge hiding into model 5 simultaneously on the innovation performance of startups. It can be found that the absolute value of the regression coefficient of entrepreneurial team psychological capital becomes smaller (from 0.268 to 0.237) and the significance level becomes lower relative to model 6 after the introduction of the mediating variable knowledge hiding, indicating that the mediating effect of knowledge hiding exists, and it is partially mediated effect, and hypothesis H5 was verified.

### 5.4. The moderating effect of organizational innovation climate

To test the moderating effect of organizational innovation climate on the relationship between knowledge sharing and innovation performance of startups and knowledge hiding and innovation performance of startups, respectively, two pairs of variables, knowledge sharing and organizational innovation climate, and knowledge hiding and organizational innovation climate, were firstly centered to reduce the effect of multicollinearity, and then regression tests were conducted, and the results are shown in Table 4. According to model 9 in Table 4, the regression coefficient of the interaction term between knowledge sharing and organizational innovation climate is 0.267 and significant at the 0.05 level, indicating that organizational innovation climate positively regulates the relationship between knowledge sharing and innovation performance of startups, and hypothesis H6 is verified. According to model 10 in Table 4, the regression coefficient of the interaction term between knowledge hiding and organizational innovation climate is −0.253 and significant at the 0.05 level, indicating that organizational innovation climate negatively regulates the relationship between knowledge hiding and innovation performance of startups, and hypothesis H7 is verified.

## 6. Conclusion and discussion

### 6.1. Research conclusion

This study aims to answer the question of whether and how psychological capital of entrepreneurial teams affects innovation performance of entrepreneurial firms. From the perspective of knowledge management, based on the survey data of 113 entrepreneurial teams in China’s provinces, this paper explores the relationship between team psychological capital and innovation performance, as well as the mediating role of knowledge sharing and knowledge hiding and the moderating role of organizational innovation climate.

Our research results show that entrepreneurial team psychological capital has a positive and significant role in promoting the innovation performance of entrepreneurial enterprises. This implies that managers should pay attention to the important role played by psychological capital in organizational innovation.

The conclusion that entrepreneurial team psychological capital is positively related to their knowledge sharing behavior and negatively related to their knowledge hiding behavior is consistent with the hypothesis, which enriches our understanding of psychological capital and knowledge management. In addition, the study confirms that knowledge sharing and knowledge hiding play a partially mediating role in the relationship between entrepreneurial team psychological capital and innovation performance of entrepreneurial firms, which suggests that in innovation management practice, the importance managers attach to the psychological capital of their teams facilitates the smooth implementation of organizational knowledge management activities, and ultimately contributes to the achievement of overall organizational goals. Finally, the moderating role of organizational innovation climate in knowledge management and innovation performance is verified, that is, the higher the organizational innovation climate is, the stronger the positive influence of entrepreneurial team knowledge sharing behavior on innovation performance of entrepreneurial enterprises, while the negative influence of entrepreneurial team knowledge hiding behavior on innovation performance of entrepreneurial enterprises is weakened.

### 6.2. Theoretical contributions

First, this paper investigates the psychological capital of entrepreneurial teams, which makes up for the deficiencies of previous studies that only focus on the psychological capital of employees, leaders, followers and entrepreneurs, and enriches the research on team psychological capital in the context of entrepreneurship. Secondly, this paper constructs a systematic research framework on the relationship between psychological capital, knowledge sharing, knowledge hiding, organizational innovation climate and innovation performance of entrepreneurial enterprises, analyzes the theoretical relationship among them, and reveals the mechanism of the role of entrepreneurial team psychological capital on innovation performance of startups. Finally, from the perspective of knowledge management, this paper discusses the impact path of psychological capital on innovation performance, and explores the mediating effects of knowledge sharing and knowledge hiding in it, which enriches the intersection research in the field of organizational psychology and knowledge management.

### 6.3. Managerial implications

Our study provides practical management insights for enterprises. On the one hand, our study shows that psychological capital of entrepreneurial teams can trigger knowledge sharing and knowledge hiding behaviors in the field of knowledge management, which provides new ideas for improving innovation performance of firms. Therefore, we suggest that enterprises should pay attention to psychological capital and clarify the role of psychological capital in innovation performance. Specifically, while focusing on individual psychological capital, enterprises should also pay attention to team psychological capital and team members’ mental health.

On the other hand, the role of the influence brought by knowledge management on innovation activities has been widely recognized by the academic community. We suggest that enterprises should conduct regular training in knowledge management, cultivate an open and mutually supportive cooperation atmosphere, encourage employees to actively participate in team cooperation, focus on collective interests, improve team members’ sense of belonging, stimulate team members’ willingness to share knowledge. Moreover, let team members understand that knowledge hiding behavior is a manifestation of mistrust among organization members, that knowledge hiding is not conducive to the realization of the overall goals of the organization, team members should trust each other and not blindly pursue the maximization of personal interests.

### 6.4. Limitations and future research direction

The research limitations of this paper are mainly reflected in three aspects: first, due to the limited number of surveyed startups and startup teams sample size, the limited sample size may have a certain degree of influence on the research results, so we hope to expand the geographical distribution and business scope of the data and samples in the future. For example, the samples of entrepreneurial enterprises and entrepreneurial teams should reasonably select entrepreneurial enterprises in eastern, central and western China. In addition, traditional industries and high-tech industries should be reasonably selected to make them broad and representative.

Second, the model in this paper only considers the moderating role of the variable “organizational innovation climate” in the influence of knowledge sharing and knowledge hiding behaviors of entrepreneurial teams on innovation performance of entrepreneurial enterprises, ignoring the role of other factors. For example, the application of digital technology can promote the flow of resources and knowledge among internal and external members of an organization, which in turn promotes the renewal of organizational knowledge and the construction of organizational capabilities. Therefore, in the context of digital competition, the influence of digital technology and other factors on innovation activities cannot be ignored, and future research can focus on the role of digital economy, digital technology, digital empowerment and other factors in the relationship between knowledge management and innovation.

Finally, the research model in this paper reveals the mechanism of the impact of team-level psychological capital on innovation performance from the perspective of knowledge management, and lacks research on the impact of different innovation types. Future research can consider the following questions: such as whether team psychological capital can promote organizational process innovation, product innovation, service innovation, business model innovation, and knowledge innovation? Are there differences in the impact of team psychological capital on different types of innovation? These problems will be the future research direction.

## Data availability statement

The raw data supporting the conclusions of this article will be made available by the authors, without undue reservation.

## Author contributions

MC was responsible for the writing—original draft, formal analysis, methodology, and conceptualization of this study. XM contributed to the manuscript writing and hypothesis model design. JX collected and screened literature, contacted channels to issue questionnaires, and communicated with participants. YL and JS participated in the manuscript writing and analyzed the data. All authors contributed to the article and approved the submitted version.
